# Cardiosphere-Derived Cells Facilitate Heart Repair by Modulating M1/M2 Macrophage Polarization and Neutrophil Recruitment

**DOI:** 10.1371/journal.pone.0165255

**Published:** 2016-10-20

**Authors:** Al Shaimaa Hasan, Lan Luo, Chen Yan, Tian-Xia Zhang, Yoshishige Urata, Shinji Goto, Safwat A. Mangoura, Mahmoud H. Abdel-Raheem, Shouhua Zhang, Tao-Sheng Li

**Affiliations:** 1 Department of Stem Cell Biology, Atomic Bomb Institute, Nagasaki University Graduate School of Biomedical Sciences, Nagasaki, Japan; 2 Department of Medical Pharmacology, Qena Faculty of Medicine, South Valley University, Qena, Egypt; 3 Department of General Surgery, Jiangxi Provincial Children's Hospital, Nanchang, Jiangxi, China; Georgia Regents University, UNITED STATES

## Abstract

Cardiosphere-derived cells (CDCs), one of the promising stem cell sources for myocardial repair, have been tested in clinical trials and resulted in beneficial effects; however, the relevant mechanisms are not fully understood. In this study, we examined the hypothesis that CDCs favor heart repair by switching the macrophages from a pro-inflammatory phenotype (M1) into a regulatory anti-inflammatory phenotype (M2). Macrophages from mice were cultured with CDCs-conditioned medium or with fibroblasts-conditioned medium as a control. Immunostaining showed that CDCs-conditioned medium significantly enhanced the expression of CD206 (a marker for M2 macrophages), but decreased the expression of CD86 (a marker for M1 macrophages) 3 days after culture. For animal studies, we used an acute myocardial infarction model of mice. We injected CDCs, fibroblasts, or saline only into the border zone of infarction. Then we collected the heart tissues for histological analysis 5 and 14 days after treatment. Compared with control animals, CDCs treatment significantly decreased M1 macrophages and neutrophils but increased M2 macrophages in the infarcted heart. Furthermore, CDCs-treated mice had reduced infarct size and fewer apoptotic cells compared to the controls. Our data suggest that CDCs facilitate heart repair by modulating M1/M2 macrophage polarization and neutrophil recruitment, which may provide a new insight into the mechanisms of stem cell-based myocardial repair.

## Introduction

Myocardial infarction (MI) induces inflammation to initiate myocardial repair [1]. This inflammatory response consists of a cascade of events; first, infiltration of neutrophils and monocytes/macrophages to clear up necrotic debris; later, extracellular matrix deposition and growth factors release; and finally, resolution of inflammation and scar formation [2]. However, excessive infiltration of the inflammatory cells into the myocardium may exacerbate heart injury and worsen post-MI remodeling, by releasing pro-inflammatory cytokines, cytotoxic mediators, and reactive oxygen species (ROS) [1, 3–5].

Macrophages are considered an important component of innate immunity and play essential roles in the inflammatory response [6]. Macrophages have two different phenotypes, the classically activated (M1) macrophages and the alternatively activated (M2) macrophages [7]. M1 macrophages display pro-inflammatory activities, while M2 macrophages have anti-inflammatory/reparative effects and contribute to resolution of inflammation, angiogenesis and tissue remodeling [6–10]. After MI, M1 macrophages keep predominant at the inflammatory phase (within 5 days after MI) and exhibit phagocytic, proteolytic, and inflammatory properties, whereas M2 macrophages dominate later (the injury-resolution phase) to promote tissue repair/remodeling [11].

Cardiosphere-derived cells (CDCs), the cardiac type of mesenchymal stem cells (MSCs), represent an attractive stem cell source for repairing an injured heart [12–16]. CDCs exhibit their functional benefits for myocardial repair largely through paracrine mechanisms [14, 17]. Previous studies have demonstrated the immunomodulation properties of MSCs from bone marrow and adipose tissue [18–21]. A recent study has also found that the therapeutic properties of CDCs are in association with the suppression of immune response [22].

In this study, we tested the hypothesis that CDCs facilitate heart repair by modulating M1/M2 macrophage polarization. Our results showed that CDCs-conditioned medium significantly promotes the macrophages to shift into a regulatory (M2) phenotype. In an acute MI model of mice, we found that the treatment with CDCs improves heart repair, accompanied by an increase in M2 macrophages and a decrease in neutrophils in the infarcted heart.

## Materials and Methods

### Animals

Adult (10-12-weeks-old) male C57BL/6 mice (CLEA Japan, Inc.) were used for the study. All experiments were approved by the Institutional Animal Care and Use Committee of Nagasaki University, and performed in accordance with the institutional and national guidelines.

### Ex vivo expansion of CDCs and fibroblast culture

Mouse CDCs were expanded using methods similar to those previously described [23]. Briefly, atria from mice were minced into small fragments and cultured as “explants” on dishes coated with 15 μg/ml fibronectin (CORNING), by using IMDM basic medium (Gibco), supplemented with 10% fetal bovine serum (HyClone), 100 units/ml penicillin G and 10 μg/ml streptomycin (WAKO, Japan). Stromal-like flat cells and phase-bright round cells emerged from the tissue fragments in 3–5 days and became confluent within 2 weeks. Twice-passaged CDCs were used for the experiments.

Mouse embryonic fibroblasts (MEFs) were purchased from company (EmbryoMax® PMEF-P3, strain CF-1, Millipore, Billerica, MA) and cultured on 0.1% (w/v) gelatin-coated culture dishes in high glucose DMEM medium (Wako, Japan), supplemented with 10% fetal bovine serum, 100 units/ml penicillin G and 10 μg/ml streptomycin.

Totally confluent CDCs or MEFs were changed with fresh medium, and conditioned media were obtained 24 hours later. All cells were cultured in a 5% CO_2_ incubator at 37°C.

### Isolation and culture of macrophages

Thioglycolate-elicited peritoneal macrophages were obtained as previously described [24]. Briefly, peritoneal macrophages were elicited by intraperitoneal injection with 3 ml of 3% sterile thioglycolate solution (Sigma-Aldrich). Four days later, macrophages were harvested by peritoneal lavage with 5 ml PBS. Freshly collected macrophages were plated in 4-well chamber culture slides (Lab-Tek, Thermo scientific Nunc) at a density of 4x10^4^/well in RPMI 1640 medium (Wako, Japan) supplemented with 10% fetal bovine serum, 100 units/ml penicillin G and 10 μg/ml streptomycin. After 2 hours of incubation, non-adherent cells were removed by washing with PBS. Adherent macrophages were cultured with CDCs- or MEFs-conditioned medium for another 3 days. The cells were fixed with 4% PFA for 10 min. After blocking, M1 and M2 macrophages were stained with the primary antibodies against CD86 (eBioscience) and, CD206 (R&D System), respectively and followed by the appropriate fluorescence-conjugated secondary antibodies. The cell nuclei were stained with 4’, 6-diamidino-2-phenylin-dole (DAPI). The positively stained cells were counted under a fluorescent microscope with 200-fold magnification. Twenty fields per section were randomly selected for quantitative counting.

### Myocardial infarction model and CDCs treatment

Myocardial infarction model was made in mice by ligating the left anterior descending coronary artery as previously described [12]. Mice were then randomly received the injection with CDCs (1x10^5^/40ul PBS), MEFs (1x10^5^/40ul PBS), or 40 ul PBS alone, into four sites around the border zone of infarction. All surgical procedures were carried out while the animals were under general anesthesia with an intraperitoneal injection of 160 mg/kg pentobarbital, and the animals remained in a supervised setting until becoming fully conscious. The animals were euthanized by severing the aorta under general anesthesia at 5 and 14 days after treatments, and the hearts were quickly washed with 5 ml cold cardioplegic solution (Mochida Pharmaceutical Co., LTD.). The ventricular tissues were embedded in OCT compound for histological analysis.

### Histological analyses

The ventricular tissues were sectioned in 5-μm sections and fixed with 4% paraformaldehyde. To evaluate the fibrotic change and tissue remodeling of the infarcted heart, Masson’s trichrome staining was done according to the manufacturer’s protocol (Sigma-Aldrich). High-resolution images were acquired with Keyence BZ-9000 fluorescence microscope and processed with Image J software: color channels were split and the infarct area was manually traced on the blue channel. Threshold adjustment and area measurement functions allowed automatic calculation of the collagen-stained fraction within the defined infarct region.

Apoptotic cells within the infarction area were detected by TACS 2 TdT-Fluor In Situ Apoptosis Detection Kit (Trevigen, Inc), according to the manufacturer’s instructions. Angiogenesis within the border zone of infarction was also evaluated by immunostaining with a primary antibody against CD31 (Abcam) and followed by the appropriate fluorescence-conjugated secondary antibody. The cell nuclei were labeled with DAPI. Terminal deoxynucleotidyl transferase dUTP nick-end labeling (TUNEL)-positive apoptotic cells and CD31-positive microvessels were viewed by microscopy (Olympus IX83, Olympus), and digital images were acquired with a DP80 camera using the cellSens software (Olympus), with 200-fold magnification. Twenty fields per section were randomly selected for quantitative counting.

To detect the macrophages and neutrophils in the infarcted area, tissue sections were fixed with 4% PFA. After blocking, the sections were incubated with primary antibodies against F4/80 (Abcam), CD206 (R&D System), CD86 (eBioscience), and NIMP-R14 (Abcam), and followed by the appropriate fluorescence-conjugated secondary antibodies. The cell nuclei were labeled with DAPI. Positively stained cells were counted as described above.

### Statistical analysis

All the results are presented as the means ± SEM. The statistical significance between two groups was determined by unpaired Students *t*-test (GraphPad Prism). Multiple comparisons were done by one-way ANOVA and followed by Turkey’s test. Differences were considered significant when p<0.05.

## Results

### CDCs significantly polarized macrophages from M1 to M2 phenotype in vitro

**Fig 1:**
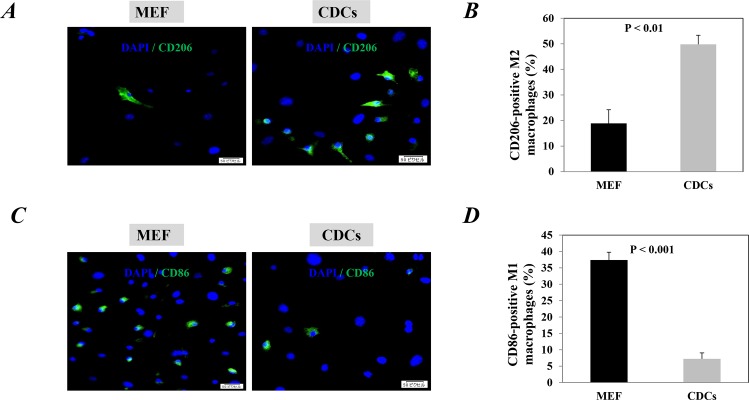
Cardiosphere-derived cells (CDCs) polarized macrophages toward M2 phenotype. Thioglycollate-activated macrophages were collected from mice and cultured with CDCs- or mouse embryonic fibroblasts (MEF)-conditioned medium for 3 days. Representative images showed the CD206-positive M2 macrophages (A) and CD86-positive M1 macrophages (C) in each group. The nuclei were stained with DAPI. Scale bar: 50 μm. Quantitative data of CD206-positive M2 macrophages (B) and CD86-positive M1 macrophages (D) are the mean± SEM from 3 independent experiments.

First, we investigated whether the CDCs have the ability to modulate macrophage polarity in vitro. Thioglycolate-activated macrophages were collected from mice and cultured with CDCs- or MEFs-conditioned medium for 3 days. Compared with the MEFs-conditioned medium, the proportion of CD206-positive M2 macrophages significantly increased after culturing with CDCs-conditioned medium (P<0.01, Fig 1A), while CD86-positive M1 macrophages significantly decreased (P<0.001, Fig 1C).

### CDCs treatment significantly facilitated the repair/regeneration of the infarcted heart

**Fig 2:**
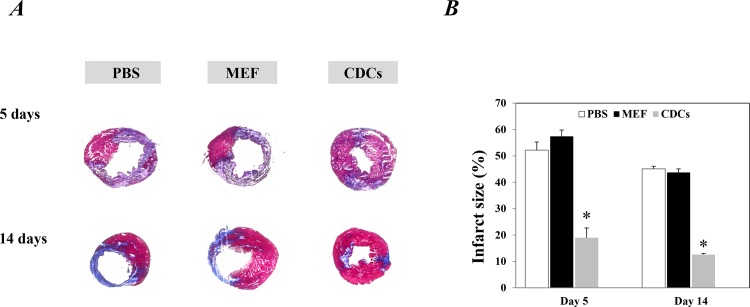
Histological analysis of the infarction size 5 and 14 days after treatments. (A) Representative images of Masson’s trichrome staining showed the infarction area (blue color). (B) Quantitative data of infarct scar size are the mean± SEM from 5 or 6 independent individuals at each time point in each group. *p<0.05 versus control groups for each time point. CDCs: Cardiosphere-derived cells; MEF: mouse embryonic fibroblasts.

**Fig 3:**
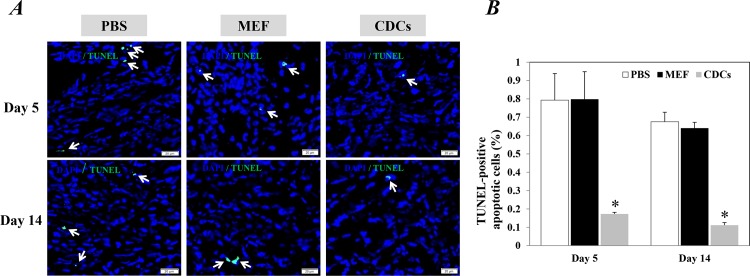
Apoptotic cells in the infarcted heart 5 and day 14 after treatments. (A) Representative images showed the TUNEL-positive apoptotic cells (green) within the infarction area. The nuclei were stained with DAPI. Scale bar: 20 μm. (B) Quantitative data of TUNEL-positive apoptotic cells are the mean± SEM from 5 or 6 independent individuals at each time point in each group. Arrows in (A) indicate positively stained cells. *P<0.05 versus control groups for each time point. CDCs: Cardiosphere-derived cells; MEF: mouse embryonic fibroblasts.

**Fig 4:**
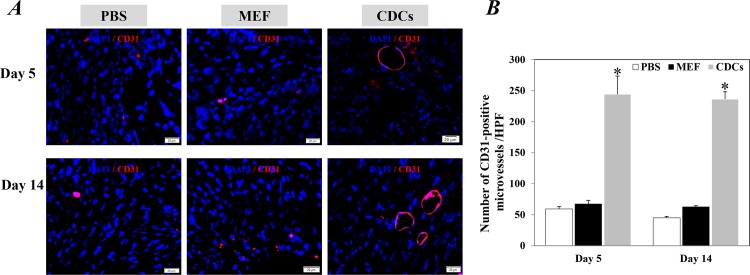
Microvessel density around the border zone of infarction 5 and day 14 after treatments. (A) Representative images showed the microvessels stained positively with CD31 antibody. The nuclei were stained with DAPI. Scale bar: 20 μm. (B) Quantitative data of microvessel density are the mean± SEM from 5 or 6 independent individuals at each time point in each group. *p<0.05 versus control groups for each time point. CDCs: Cardiosphere-derived cells; MEF: mouse embryonic fibroblasts.

Compared with controls, Masson’s trichrome staining revealed that the implantation with CDCs significantly reduced infarct size at 5 and 14 days after treatments (P<0.05, Fig 2A). Significantly fewer apoptotic cells within the infarcted hearts were also detected in mice injected with CDCs compared to the controls at 5 and 14 days after treatments (P<0.05, Fig 3A). Furthermore, myocardial neovascularization evaluated by immunostaining with CD31 showed that the density of microvessels around the border zone of infarction was significantly higher in the infarcted heart of mice received CDCs compared with controls (P<0.05, Fig 4A). These data confirmed the potency of CDCs for myocardial repair/regeneration in an acute infarcted heart.

### CDCs treatment increased M2 macrophages and reduced the neutrophils in the infarcted heart

**Fig 5:**
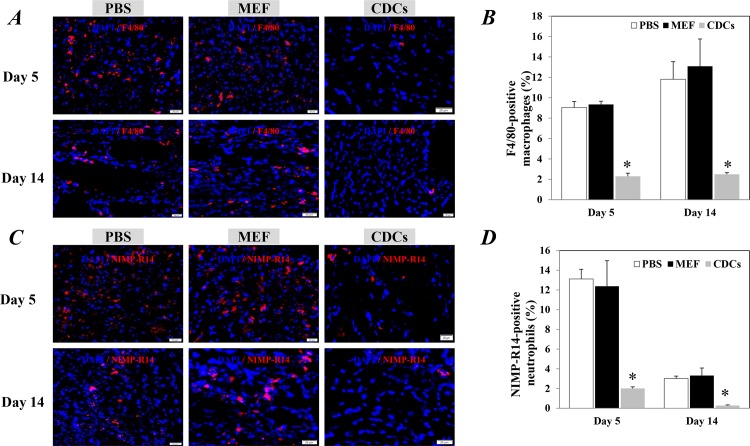
The recruitment of the inflammatory cells in the infarcted heart 5 and day 14 after treatments. Representative images showed the whole macrophages (F4/80 positive, A) and NIMP-R14-positive neutrophils (C) within the infarction area. The nuclei were stained with DAPI. Scale bar: 20 μm. Quantitative data of F4/80-positive macrophages (B) and NIMP-R14 positive-neutrophils (D) are the mean± SEM from 5 or 6 independent individuals at each time point in each group. *p<0.05 versus control groups for each time point. CDCs: Cardiosphere-derived cells; MEF: mouse embryonic fibroblasts.

To identify the ability of CDCs for regulating the inflammatory response, we investigated the levels of macrophages and neutrophils in the infarcted hearts 5 and 14 days after treatments. The number of whole macrophages positively stained by F4/80 was significantly lower in the infarction area of hearts treated with CDCs compared to the controls (P<0.05, Fig 5A). The number of NIMP-R14-positive neutrophils in the infarction area was also significantly lower in hearts treated with CDCs compared with controls (P<0.05, Fig 5C).

**Fig 6:**
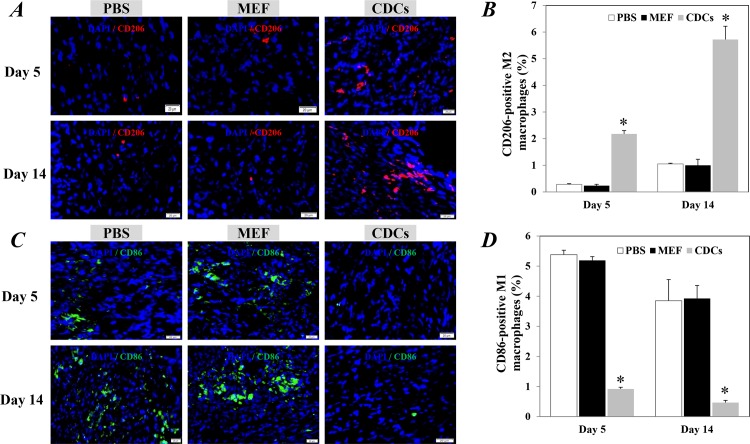
The M1 and M2 macrophages in the infarcted heart 5 and day 14 after treatments. Representative images showed the CD206-positve M2 macrophages (A) and CD86-positive M1 macrophages (C) within the infarction area. The nuclei were stained with DAPI. Scale bar: 20 μm. Quantitative data of CD206-positve M2 macrophages (B) and CD86-positive M1 macrophages (D) are the mean± SEM from 5 or 6 independent individuals at each time point in each group. *p<0.05 versus control groups for each time point. CDCs: Cardiosphere-derived cells; MEF: mouse embryonic fibroblasts.

By using specific primary antibodies against CD86 and CD206, we further distinguished the macrophages into M1 and M2 subsets, respectively. Compared with controls, the CD206-positive M2 macrophages were detected significantly higher in the infarcted hearts at 5 days after the treatment with CDCs (P<0.05, Fig 6A). The M2 macrophages were even further increased in the CDCs-treated mice at 14 days after treatment (P<0.05, Fig 6A). In contrast, the CD86-positive M1 macrophages were significantly reduced in the infarcted hearts of mice received CDCs compared with controls, at 5 and 14 days after treatment (P<0.05, Fig 6C).

## Discussion

Within the last decade, stem cells, especially the CDCs have been proved to be beneficial for myocardial repair by pre-clinical and clinical studies [25–28], but the complex mechanisms are still investigated to be further understood. In the present study, we found that CDCs-conditioned medium polarized macrophages from a pro-inflammatory (M1) phenotype into an anti-inflammatory (M2) phenotype in vitro. For in vivo experiments, the implantation of CDCs into the infracted myocardium reduced the levels of overall macrophages and neutrophils, but increased the proportion of M2 macrophages. Our data suggest that CDCs may facilitate myocardial repair by regulating M1/M2 macrophage polarization and neutrophil recruitment.

Macrophages are essential mediators of the inflammatory response and play an important role in the initiation and resolution of inflammation. After MI, many macrophages infiltrate into the infarcted heart. It has been demonstrated that the healing of infarcted myocardium is supposed to occur through macrophage transition; a conversion from a pro-inflammatory (M1) phenotype to an anti-inflammatory (M2) phenotype [29]. However, enhanced infiltration of the inflammatory cells into the myocardium may exaggerate heart injury and worsen post-MI remodeling [29, 30]. Panizzi et al. reported that the accentuation, prolongation or expansion of the inflammatory response following MI, impairs the healing of the infarction in apoE-/- mice [30]. This suggests that the modulation of the inflammatory response may improve the healing and attenuate LV remodeling of the injured heart [30].

CDCs, one of the most promising stem cell sources for heart repair have been characterized as a cardiac type of mesenchymal stem cells [12–16]. The immunomodulation property of MSCs has been demonstrated by previous studies [18–21]. Therefore, it is not surprising about the M1/M2 macrophage polarization after culturing macrophages with the CDCs-conditioned medium. Using an acute MI model of mice, the efficiency of CDCs for repairing a damaged heart was well evidenced by; attenuation of apoptosis, promotion of angiogenesis, and reduced infarct size. Interestingly, the treatment with CDCs also significantly reduced the recruitment of neutrophils, but especially increased the M2 macrophages in the infarcted myocardium.

Shiraishi M et al. [31] have very recently confirmed the essential role of M2 macrophages in myocardial repair. By using Trib1-/- mice, in which the M2 macrophages in various organs were severely reduced without affecting other inflammatory cells, including the M1 macrophages [32]. They have demonstrated that the specific depletion of M2 macrophages resulted in impairment of cardiac function and increased the risk of cardiac rupture [31]. A recent study by Hu et al. [33] have also proved the critical role of scavenger receptor A (SR-A); a homologous to CD206 that is strongly expressed in M2 macrophages, in repairing the infarcted myocardium [33, 34]. They have found that the depletion of SR-A in mice resulted in an increase in M1 macrophages with a subsequent decrease in M2 macrophages, and impairment of myocardial recovery following MI [33]. This suggests that M2 macrophages may play an important role in the repair of the myocardial infarction and the attenuation of post-MI remodeling.

Based on the findings from past studies and our data, we have found that the increase in M2 macrophages by CDCs treatment may facilitate, at least partially the repair of the infarcted heart. Our results of the in vitro study showed that CDCs have the capacity to modulate M1/M2 macrophage polarization. This suggests that the treatment with CDCs may increase M2 macrophages and modulate the inflammatory response by releasing soluble factors. This has once again indicated that the beneficial effects of CDCs for myocardial repair through paracrine mechanisms, rather than direct regeneration [14, 17].

Although our results provided a novel insight into the mechanisms of stem cell-based myocardial repair, several limitations should be mentioned. First, our study was primarily designed to search whether CDCs have an immunomodulatory effect or not. So, we did not try to understand how the CDCs regulated the macrophages into M2 phenotypes and reduced neutrophils recruitment. Based on past studies [35–45], complex mechanisms will be involved in CDCs mediating M1/M2 switch and neutrophil recruitment. Second, although CDCs-conditioned medium mediated M1/M2 polarization in vitro, we did not provide evidence on the macrophage phenotypic switch in vivo. The treatment with CDCs may improve the survival and recruitment of M2 macrophages in the infarcted heart, however, it is still unclear whether and how the increased number of M2 macrophages actually contributed to myocardial repair. Otherwise, we did not measure the viable myocardium and the cardiac function, and the apoptotic cells were only identified by TUNEL staining. Further experiments are needed to explore how the CDCs regulate M1/M2 polarization and decrease neutrophil recruitment.

In summary, data from the present study showed that CDCs could mediate a switch of macrophages into a regulatory anti-inflammatory (M2) phenotype, which may provide a novel insight into the mechanisms of CDCs for myocardial repair. The modulation of the inflammatory response by stem cell therapy will be of great significance in enhancing the treatment of heart failure, especially in patients with excessive and prolonged inflammatory responses.
