# Correction: Cardiosphere-Derived Cells Facilitate Heart Repair by Modulating M1/M2 Macrophage Polarization and Neutrophil Recruitment

**DOI:** 10.1371/journal.pone.0171892

**Published:** 2017-02-06

**Authors:** Al Shaimaa Hasan, Lan Luo, Chen Yan, Tian-Xia Zhang, Yoshishige Urata, Shinji Goto, Safwat A. Mangoura, Mahmoud H. Abdel-Raheem, Shouhua Zhang, Tao-Sheng Li

There are errors in the Funding section. The correct funding information is as follows: This study was partially supported by a Grant-in-Aid from the Ministry of Education, Science, Sports, Culture and Technology, Japan, and partially supported by the National Natural Science Foundation of China (81460118-Dr. Shuohua Zhang). Dr. Al Shaimaa Hasan is supported by the joint supervision mission scholarship from the Ministry of Higher Education, Egypt. The funders played no role in the study design, data collection and analysis, decision to publish, or preparation of the manuscript.

There is a sentence missing after the fourth sentence of the third paragraph of the Introduction. The sentence should read: Furthermore, it has been recently demonstrated that CDCs limit acute injury by polarizing an effector macrophage population within the heart.

A reference is omitted from the fourth sentence of the third paragraph of the Introduction, the second sentence of the fourth paragraph of the Introduction, and the second sentence of the third paragraph of the Discussion.

The reference is: de Couto G, Liu W, Tseliou E, Sun B, Makkar N, Kanazawa H, Arditi M, Marbán E. Macrophages mediate cardioprotective cellular postconditioning in acute myocardial infarction. J Clin Invest. 2015;125:3147–62. doi: 10.1172/JCI81321.

The fourth sentence of the third paragraph of the Introduction should read: Furthermore, it has been recently demonstrated that CDCs limit acute injury by polarizing an effector macrophage population within the heart (de Couto et al., 2015).

The second sentence of the fourth paragraph of the Introduction should read: Similar to previous study (de Couto et al., 2015), results showed that CDCs-conditioned medium significantly promotes the macrophages to shift into a regulatory (M2) phenotype.

The second sentence of the third paragraph of the Discussion should read: The immunomodulation property of MSCs has been demonstrated by previous studies, and a recent study has already demonstrated the potential role of CDCs for polarizing macrophages [18–21] (de Couto et al., 2015).

There are errors in the Author Contributions. The correct contributions are: Conceptualization: AH MAR SZ TL. Data curation: AH TL. Formal analysis: AH LL CY TZ YU SG MAR SMSZ TL. Funding acquisition: SZ TL. Investigation: AH LL CY TZ YU SG MAR SMSZ TL. Methodology: AH MAR SZ TL. Project administration: TL. Resources: AH LL CY TZ YU SG SZ TL. Software: LL CY YU SG. Supervision: TL. Validation: MAR SZ TL. Visualization: AH LL CY TZ YU SG MAR SMSZ TL. Writing–original draft: AH TL. Writing–review& editing: AH TL.
